# Efficacy of the Enteroadsorbent Silicol®gel in Adults with Irritable Bowel Syndrome Subtypes IBS-D or Mixed: Observational Open-Label Study

**DOI:** 10.1155/2023/3432763

**Published:** 2023-12-16

**Authors:** Gordon Crawford, Rory Taylor, David Young, Chris G. Hatton

**Affiliations:** ^1^CPS Research, McCafferty House, 99 Firhill Road, Glasgow, UK G20 7BE; ^2^Department of Mathematics and Statistics, University of Strathclyde, 26 Richmond Street, Glasgow, UK G1 1XH; ^3^NHS, Greater Glasgow and Clyde, 1055 Great Western Road, Glasgow, UK G12 0XH; ^4^FW Medical Ltd, West of Scotland Science Park, Kelvin Campus, Block 6, Glasgow, UK G20 0SP

## Abstract

**Background:**

Irritable bowel syndrome (IBS) is a common chronic gut-brain interaction disorder with limited effective treatment options. Intestinal adsorbents have a high adsorption capacity for gut irritants and may provide nonpharmacological alternatives.

**Objectives:**

This post marketing study is aimed at providing up-to-date evidence to support the safety and efficacy in normal use of an established medical device for IBS treatment.

**Methods:**

In this open-label, observational study, adults with IBS with predominant diarrhoea (IBS-D) or IBS with mixed bowel habits (IBS-M), according to Rome IV criteria, received 4 weeks of treatment with the enteroadsorbent Silicol®gel, a CE-certified, licenced, medical device containing colloidal silicic acid. Eligible participants were assessed at baseline (visit 1; in-clinic) and after 1 (visit 2; telephone), 2 (visit 3; telephone), and 4 (visit 4; in-clinic) weeks of treatment. The primary endpoint was the proportion of participants with an overall reduction in the IBS severity scoring system (IBS SSS) > 50, representing clinically meaningful improvement. Key secondary endpoints were a reduction in common IBS symptoms and improved quality of life (QoL).

**Results:**

Among the 67 treated participants (IBS-D: 37; IBS-M: 30), 65 completed the study. At visit 4, 83.6% (56/67) of participants achieved a reduction in IBS SSS > 50. The mean (standard deviation [SD]) IBS SSS was 323.4 (55.7) at visit 1 and 160.3 (90.3) at visit 4 (overall change: -163.1 (101.7); 95% confidence interval [CI] 138.3, 187.9, *p* < 0.001). Compared with visit 1, significant reductions in the severity of all key IBS symptoms and overall improvement in QoL were observed at visit 4 (*p* < 0.001), with improvements observed from visits 1 and 2.

**Conclusions:**

In this open-label study of participants with IBS-D and IBS-M, Silicol®gel provided clinically significant improvement in IBS symptoms, demonstrating that enteroadsorbents may be clinically beneficial in this population.

## 1. Introduction

Irritable bowel syndrome (IBS) is a common chronic disorder of gut-brain interaction (DGBI), formerly classified as a functional gastrointestinal (GI) disorder [1, 2]. Key symptoms of IBS include abdominal pain, altered bowel habit, and abdominal bloating or distention. IBS may be characterised based on symptomatology as IBS with predominant diarrhoea (IBS-D), IBS with predominant constipation (IBS-C), IBS with mixed bowel habits (IBS-M), or IBS unclassified (IBS-U) [1, 2].

The diagnosis of IBS is based on the Rome IV criteria—a set of symptom-based diagnostic criteria developed by the Rome Foundation [2]. Using these criteria, a large multinational study reported a global IBS prevalence for an Internet survey sample (*N* = 54,127 individuals) of 4.1%; among these, the Rome IV subtypes were 28.7% IBS-D, 32.4% IBS-C, 32.4% IBS-M, and 6.5% IBS-U [3]. The prevalence is higher among women, and affected individuals are typically younger adults [2, 3].

IBS is associated with several comorbidities, including somatic pain syndromes (fibromyalgia, chronic fatigue, and chronic pelvic pain), gastrointestinal disorders (gastroesophageal reflux and dyspepsia), and psychiatric disorders (depression, anxiety, and somatisation) [4–10]. Consequently, IBS imposes a significant burden on individuals' quality of life (QoL) and negatively impacts daily functioning, including social functioning and the ability to work, particularly among people with IBS-D and IBS-M [11–14]. Interference with daily activities is significantly more prevalent among participants with IBS-D and IBS-M than among those with other IBS subtypes [14]. This may be attributable to increased bowel frequency, which may limit individuals' social functioning; indeed, bowel urgency is reported to be one of the most bothersome symptoms in IBS-D [14–16]. High levels of impairment in daily functioning are more common in people with IBS and comorbid anxiety, depression, and panic disorder [11]. The economic burden of IBS is also considerable, with one study published in 2019 estimating annual direct and indirect costs due to IBS in Italy of up to €8 billion, while in the United Kingdom, annual direct healthcare costs of £1.3 billion have been estimated for IBS using the Rome IV criteria [17, 18].

Despite the widespread prevalence of this debilitating condition, there is a lack of effective treatment options available to people with IBS, and a 2016 survey of approximately 3000 individuals reported that only 21% felt confident their IBS symptoms were under control and 34% felt that none of their symptoms were under control [19]. Initial symptom management typically involves lifestyle and dietary interventions (e.g., regular exercise, healthy diet, and reducing dietary fibre); however, pharmacotherapy, such as antispasmodics, antidiarrhoeals, and laxatives, may be required to manage symptoms of abdominal pain, diarrhoea, and constipation, respectively; behavioural and psychological treatments may also be used [1, 20]. People with IBS frequently use multiple medications/treatments to control their IBS symptoms, and 62% of individuals with IBS in one survey reported using two or more medications on a regular basis [19].

Intestinal adsorbents, such as dioctahedral smectite and silicic acid, that have historically been used for the adsorption of toxins and poisons offer nonpharmacological alternatives to manage the symptoms of IBS [21–25]. As the activity of these ingredients is due to a physical effect, they are classified as medical devices. Their mode of action is not completely understood and is considered to be related to their high adsorption capacity for irritant substances found in the gut (e.g., reactive enterotoxins, pathogens, and bile acids) and a potential mucoprotective effect [21–23, 26–31]. Until recently, few contemporary studies have been published on intestinal adsorbents in the treatment of IBS [21–23, 32]. These randomised, placebo-controlled studies showed that enteroadsorbents provided to individuals with IBS improved symptom control compared with placebo [21–23, 32].

Silicol®gel (Silicol GmbH, Germany) is an established CE-marked intestinal adsorbent comprising an oral gel formulation containing colloidal silicic acid approved for the relief of gastrointestinal disorders, including IBS. Silicolgel has been marketed in the UK and European markets for over 10 years [33]. This study was designed as an up-to-date investigation of the efficacy and safety of this established medical device in individuals with IBS-D and IBS-M, according to Rome IV criteria [2], and forms part of the periodic safety update that is required for medical devices [34].

## 2. Materials and Methods

### 2.1. Study Design and Recruitment

This post marketing study was designed to support the safety and efficacy of silicolgel, an established medical device, in normal usage as per the approved dosage and indications. Therefore, silicolgel continuous treatment was limited to a maximum 4-week duration in line with the medical device licence, and this was an open-label study.

Primary analyses compared IBS SSS baseline, pretreatment (visit 1—in clinic), and IBS SSS after 4 weeks of treatment with silicolgel (visit 4—in clinic).

Participants were also telephone interviewed by a study nurse after 1 and 2 weeks of treatment (visit 2 and visit 3), primarily to ensure correct product use and encourage product compliance. However, we believed that due to the mode of action of silicolgel enteroadsorbent, many sufferers would begin to feel some improvement in symptoms before the end of the 4-week treatment period. Therefore, during visit 2 and visit 3 telephone interviews, participants were asked to assess the severity of IBS symptoms and QoL using the same questionnaires used throughout the study.

In this open-label, single-centre, prospective, observational study, CPS Research—an independent clinical trial specialist—was responsible for study recruitment and management, with independent oversight by Klinikos Ltd, a clinical trial service organisation.

Participants were recruited by CPS using their established recruitment model; this involved local community advertising (e.g., GP practices and community magazines), social media advertising (Facebook/Instagram), and a private research database.

Those responding to advertising/social media completed an online questionnaire which asked the following: how did they hear about this IBS study; age; gender; pregnancy; bowel habits; do they suffer from diarrhoea, abdominal pain, bloating, and flatulence; and a history of inflammatory bowel disease, bowel cancer, or coeliac disease. Potential participants then had a detailed telephone interview with a CPS study nurse. This covered details about bowel habits, IBS, and other GI symptoms as well as background medical history and current medications.

**Figure 1:**
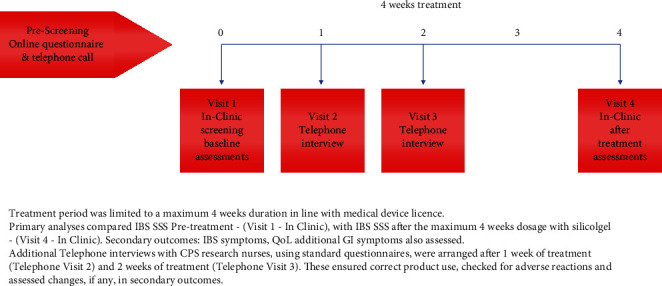
Study design.

Subjects who met prescreening criteria were invited for formal in-clinic screening (see Figure 1).

Visit 1 in-clinic screening included full Rome IV questionnaires with Bristol Stool Chart to confirm IBS diagnosis and classification, as well as full IBS SSS questionnaires, as per Francis et al. [35].

### 2.2. Funding

The study was funded by FW Medical Ltd, the owner and distributor of silicolgel. FW Medical had no direct involvement in the study. The study complied with the principles of the Declaration of Helsinki; all participants provided written informed consent.

### 2.3. Ethics Approval

This post marketing study was a safety and efficacy study, in normal use, to provide up-to-date technical support as required by MDR Medical Devices regulations. In line with local requirements determined by the health authority, there was no research ethics committee approval required for this postmarketing surveillance study on a licenced medical device being used for its intended purpose.

### 2.4. Study Participants

Participants were adults aged 18–65 years, with a BMI of 18–39 kg/m^2^, an irritable bowel syndrome severity scoring system (IBS SSS) of 150–450 (representing people across the range of IBS symptom severity—mild, moderate, and severe cases) [35], and IBS-D or IBS-M as defined by the Rome IV criteria and classification [2].

Note: candidates with IBS-C (predominantly constipation) were excluded from this trial as enteroadsorbents such as silicolgel are antidiarrhoeal and could exacerbate constipation.

Participants were also required at visit 1 to have two or more key IBS symptoms (diarrhoea, bloating, abdominal pain/cramping, or flatulence) rated 3 or more on a visual analogue scale (VAS) of 0 to 10. Participants had to be willing to maintain a consistent diet, lifestyle, and alcohol intake during the study period.

Exclusion criteria were as follows: significant acute or chronic comorbidity as determined by the investigator, inflammatory bowel disease, bowel cancer, coeliac disease, major abdominal surgery, recent use of antibiotics or corticosteroids, pregnancy or breastfeeding, previous use of silicolgel or enterosgel, uncontrolled or unstable major psychiatric disorder, planned start of any new medications during the study, subjects deemed unlikely to comply with study requirements, and recent participation in an interventional clinical trial. For full exclusion criteria, see Table 1.

A full list of all concomitant medications, prescribed and self-medicated, including IBS and intestinal medicines, was recorded before visit 1 and after treatment visit 4 (see Table 2).

### 2.5. Study Treatment (See Figure 2)

Silicolgel contains colloidal silicic acid (3.5% silicon dioxide in 100 mL silicic acid gel) and is a CE-marked (CA in the UK) class IIa medical device, according to MDD 93/42/EEC.

CE (CA) marking is mandatory for certain product groups intended for sale within the European Union/UK. Class IIa devices represent low to medium risk and refer to devices installed or remaining within the body for only 60 minutes to 30 days. Regulatory requirements include the annual auditing of the technical file by an accredited, third-party-notified body. The annual audit covers all technical, quality, safety, and efficacy documentation for both the device and the manufacturer.

Silicolgel is not absorbed into the bloodstream and works purely by physical rather than pharmacological means, acting locally in the gut. Silicolgel provides an extensive surface area built from silicon hydroxy end-groups, allowing a high adsorption capacity for irritant substances in the gastrointestinal tract.

Participants received 4 weeks of treatment with silicolgel, as per the permitted regulatory guidelines for continuous usage. Silicolgel was provided to participants in standard 15 mL branded sachets. Participants were instructed to take one sachet, either undiluted or diluted with water, three times daily, at least 1 hour before meals, allowing at least a 1-hour gap between taking silicolgel and taking medications.

### 2.6. Study Endpoints

The primary endpoint was the proportion of participants (i.e., “responders”) with an overall reduction in the IBS severity scoring system (IBS SSS) of >50 from visit 1 (baseline) to visit 4, following 4 weeks of treatment. A change of this magnitude has been shown to reliably indicate improvement in IBS symptoms [35] and has been used in this study as a means of measuring clinically meaningful improvement.

Secondary endpoints included a reduction in key IBS symptoms (diarrhoea, bloating, abdominal pain/cramping, and flatulence), an improvement in quality of life, and safety and tolerability, monitored throughout the study. In addition to in-clinic assessments at visits 1 and 4, these secondary endpoints were also recorded via telephone visits after 1 week of treatment (visit 2) and 2 weeks of treatment (visit 3). Prior marketing experience indicates that many silicolgel users feel improvement in IBS symptoms after only 1-2 weeks of treatment, and early improvement in IBS symptoms is likely to encourage continued treatment for the full duration.

Changes in additional gastrointestinal symptoms (heartburn, nausea, and vomiting) that sometimes coexist in individuals with IBS were also assessed before and after treatment.

### 2.7. Assessments

#### 2.7.1. IBS SSS (IBS Severity Scoring System)

The IBS SSS developed by Francis et al. [35] is a validated tool for the assessment of IBS severity that is accepted as an appropriate endpoint in clinical research by regulatory agencies [36] and commonly used in clinical studies of IBS [32, 37–40].

Part 1. The IBS severity score system is a composite score of abdominal pain, number of days with abdominal pain, bloating/distension, satisfaction with bowel habits, and IBS-related quality of life. Each measure is rated from 0 to 100, with a total possible score of 0-500. Scores of <75 are considered to indicate remission, scores between 75 and 174 indicate mild IBS, scores of 175 to 299 indicate moderate IBS, and scores over 300 indicate severe IBS. An overall reduction in the IBS SSS score of >50 is considered to represent a clinically meaningful improvement in disease severity.

A reduction in IBS SSS of greater than 50 was the primary endpoint for this study. A reduction in the mean IBS SSS after treatment was also a primary endpoint assessment.

Full IBS SSS questionnaires (Francis et al. [35]) were used in-clinic at baseline before treatment (visit 1) and after 4 weeks of treatment (visit 4). Participants with a reduction in IBS SSS > 50 after 4 weeks of treatment with silicolgel (visit 4) were “responders,” while participants with a reduction ≤ 50 were “nonresponders.”

Part 2. Other IBS data include outcomes such as bowel habits: frequency and quality of bowel motions, pain and bowel motions, and also work and IBS.

Full Part 2 data was collected, but the majority of this data is not reported in this manuscript as it was not a primary or secondary endpoint. However, the frequency of bowel motions is an important quality of life factor in many IBS sufferers; therefore, bowel frequency before and after treatment is reported (see Table 3).

#### 2.7.2. IBS Symptoms

Participants rated the severity of four key symptoms (diarrhoea, bloating, abdominal pain/cramping, and flatulence) on a 10-point VAS at visits 1, 2, 3, and 4; ratings were based on symptom severity “today” and over the “last 7 days.”

#### 2.7.3. Quality of Life (QoL)

Participants rated their QoL before, during, and after treatment. A simplified QoL questionnaire comprised three questions to evaluate (i) the overall effect of IBS on QoL, (ii) the extent to which IBS impacted normal activities, and (iii) the effect of IBS on negative feelings such as depression/anxiety. Each question was rated on a 5-point scale, similar to that used in the Irritable Bowel Syndrome Quality of Life (IBS-QoL) questionnaire [41].

#### 2.7.4. Additional Gastrointestinal Symptoms

Participants also rated the frequency and severity of additional gastrointestinal symptoms that sometimes coexist in individuals with IBS (heartburn, nausea, and vomiting) using a 10-point VAS. Frequency and severity were assessed before (visit 1) and after (visit 4) treatment.

#### 2.7.5. Other Assessments

Additional assessments, such as the patient's global impression and the patient's overall assessment of silicolgel efficacy, were performed. Adverse events were monitored and documented in the Case Report Form during every scheduled visit.

### 2.8. Statistical Analyses

One of the primary purposes of this post marketing study was to update the technical file for this established medical device in line with the European Medical Device Regulation. A sample size of 60 participants completing was selected as being sufficient to meet this requirement, although we were also able to consider data from an unpublished silicolgel study from 1996.

Due to a smaller-than-anticipated rate of dropouts, there were 65 study completers from 67 ITT.

A paired *t*-test was performed to analyse the reduction in IBS SSS. Variables measured over time were compared using the Friedman tests. Where evidence of a difference was shown, Wilcoxon's tests were used for post hoc comparisons between time points. All *p* values were unadjusted for multiple comparisons. Results were reported as *p* values with approximate 95% confidence intervals to reflect the effect size of silicolgel on outcome measures. All analyses were conducted using Minitab (version 18) with a 5% significance level.

An analysis of IBS SSS was done for all participants who received at least one dose of silicolgel. For missing data (*n* = 2 participants at visits 3 and 4), values from visit 2 were used. An analysis of additional gastrointestinal symptoms was reported, but there was no imputation for missing data.

## 3. Results

### 3.1. Study Participants

**Figure 3:**
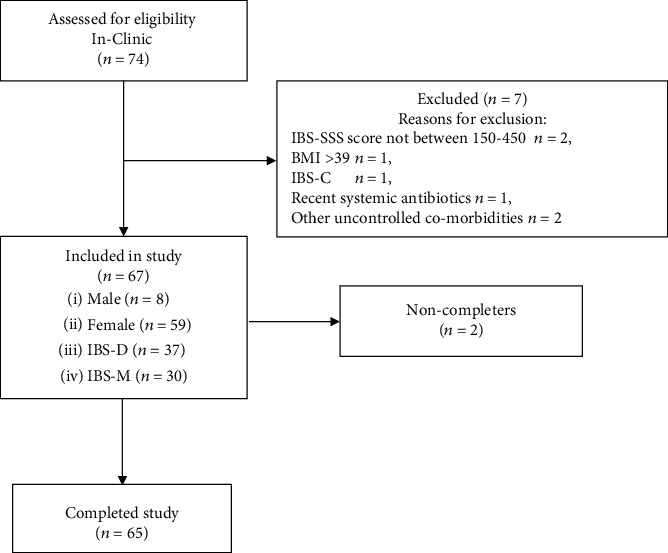
Participant disposition silicolgel study.

Following the initial online questionnaire and telephone prescreening of subjects by research nurses, 74 attended in-clinic screening (visit 1), with 7 screened out at this stage (see Figure 3).

Among the 67 participants enrolled in the study, 65 completed the study and 2 discontinued before completing the 4-week study period. Eight of the 67 participants were male and 59 were female, with 37 having IBS-D and 30 having IBS-M. The mean (range) age and BMI were 40.9 (20–64) years and 27.7 (18.6–39.0) kg/m^2^.

Baseline characteristics of the study population *N* = 67 are shown (see Table 4).

### 3.2. IBS Severity Scoring System (IBS SSS)

After 4 weeks of treatment, 83.6% of participants (56/67) achieved the primary endpoint, with a reduction in IBS SSS > 50.

**Figure 4:**
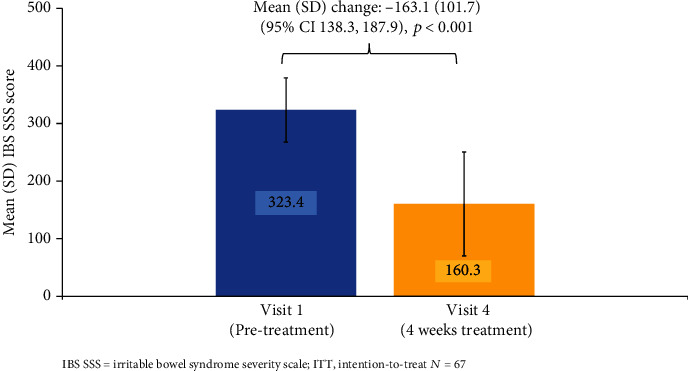
Mean IBS SSS scores before (visit 1) and after (visit 4) treatment.

The mean (SD) IBS SSS was significantly reduced from 323.4 (55.7) at visit 1 to 160.3 (90.3) at visit 4 (overall (SD) change: -163.1 (101.7); 95% CI 138.3, 187.9, *p* < 0.001) (see Figure 4).

This represents a transition in the IBS SSS questionnaire classification from severe IBS to mild IBS [35].

Among the 65 study completers, IBS SSS change > 50 was observed in 87.7% (mean [SD] IBS SSS: 323.3 (56.0) at visit 1 and 154.2 (86.7) at visit 4; overall change: -168.1). Among the 56 responders, the mean (SD) IBS SSS changed from 332.1 (visit 1) to 144.2 (visit 4; overall change: -187.9).

Nine participants (13.4%) were nonresponders with IBS SSS reductions of <50 from baseline. One participant had a change of -50, and 1 participant had a minor increase in score from 268 to 272 (not clinically significant).

IBS SSS data for both IBS type D and IBS type M subgroups was consistent with the overall total cohort data (see Table 5).

Data for Part 2 of the IBS SSS questionnaires were consistent with those from Part 1 and showed improvements at visit 4 in other IBS parameters, including a reduction in the frequency of bowel motions. Maximum frequency of bowel motions before treatment: mean [standard deviation] 4.17 per day [2.19] significantly reduced after treatment to a mean of 2.74 per day [1.65], *p* < 0.001. The minimum frequency of bowel motions was reduced from a mean of 1.56 per day [1.29] to 1.12 per day [0.91], *p* < 0.03. Similar reductions in maximum stool frequency per day were seen by both IBS-D and IBS-M participants (see Table 3).

### 3.3. Change in IBS Symptoms

Four key IBS symptoms, diarrhoea, bloating, abdominal pain/cramping, and flatulence, were assessed throughout the duration of the study using a 0 to 10 rating scale, where 0 = “Doesn't bother me at all” to 10 = “As bad as I can imagine.”

**Figure 5:**
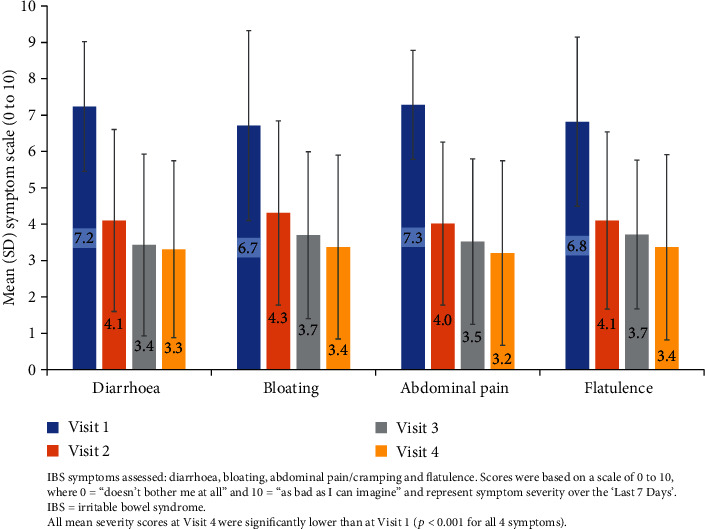
Mean severity scores of 4 key IBS symptoms over the duration of the study.

A reduction in mean severity scores was progressive across the duration of treatment, with reductions at weeks 1 and 2 seen for all four symptoms (see Figure 5).

Participants' mean severity ratings of these key IBS symptoms ranged between 6.7 and 7.3 at visit 1 before treatment. After 4 weeks of treatment (visit 4) and across all four symptoms, the mean symptom severity scores for the “last 7 days” were significantly lower (*p* < 0.001 for all symptoms), and mean scores had dropped to between 3.2 and 3.4 (see Table 6).

Similar reductions in these key IBS symptoms before and after treatment were seen for both IBS-D and IBS-M participants.

The results obtained using the symptoms data for “today” were consistent with those observed for the “last 7 days” (results not presented).

### 3.4. Quality of Life

Overall improvement in QoL, as measured with the three-question QoL questionnaire, was observed throughout the duration of the study.

Across all three questions, there was a significant improvement in mean QoL scores after treatment (visit 4) compared to before (visit 1), *p* < 0.001 for all (full data available on request).

**Figure 6:**
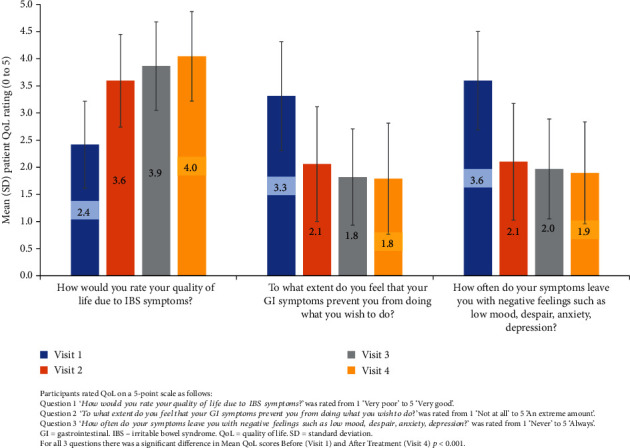
Mean (SD) quality of life ratings for the duration of the study.

The mean QoL scores also showed progressive improvements through visits 2, 3, and 4, compared with visit 1 treatment (see Figure 6).

### 3.5. Change in Additional Gastrointestinal Symptoms: Heartburn, Nausea, and Vomiting

Not all participants suffered from these additional symptoms. However, at visit 4, after 4 weeks of treatment, significant reductions in the mean scores for both frequency of suffering and severity of suffering for all these 3 additional gastrointestinal symptoms were recorded compared with visit 1 before treatment (full data available on request).

### 3.6. Other Assessments

Data for other assessments, such as the patient's global impression of their IBS and patient assessment of silicolgel efficacy, support the main findings showing improvement in symptoms after silicolgel treatment, and the majority of participants found the product easy to use. These data have not been included in the current manuscript as these parameters were not key study endpoints.

### 3.7. Safety and Tolerability

Silicolgel was well tolerated. During the study, 47 adverse events (AEs) were reported by 34 participants (see Table 7). The majority of AEs were considered not causally related to silicolgel. The 11 AEs considered possibly or probably causally related to silicolgel were all gastrointestinal, categorised as mild, and required no intervention. These gastrointestinal symptoms may be symptoms of the underlying pathology and are thus not unexpected in this type of study. There were two serious AEs reported, both of which were deemed unrelated to silicolgel; these included one event of optic neuritis and one event of severe headaches, which were present before the study commenced but were not declared by the participant during screening. Both participants dropped out after study visit 3 (data from visits 1 and 2 were included).

## 4. Discussion

This open-label, observational study demonstrates that under conditions resembling normal usage, silicolgel provides symptom improvement in participants with IBS-D and IBS-M subtypes that comprise approximately two-thirds and four-fifths of people with IBS in a global and a UK-based study of IBS using Rome IV criteria, respectively [3, 18].

### 4.1. IBS SSS

The primary study endpoint of a reduction in IBS SSS of >50 was achieved by the majority of participants (83.6%) and represented a clinically significant improvement in participants' IBS symptoms [35]. The observed mean reduction in IBS SSS (323.4 at visit 1 to 160.3 at visit 4; a change of -163.1) represented a change from severe to mild IBS, as per the Francis et al. scale [35]. This is a very high response rate and likely includes a placebo-effect response. However, this primary endpoint, which assessed IBS status by a reduction in IBS SSS, is supported by secondary outcomes: a significant reduction in the severity of four key IBS symptoms, a significant improvement in QoL ratings, and a significant reduction in the severity and frequency of other gastrointestinal symptoms (heartburn, nausea, and vomiting), all after 4 weeks of treatment.

### 4.2. Key IBS Symptoms

Significant improvements in symptom severity were observed in this silicolgel study across all four key IBS symptoms (diarrhoea, bloating, abdominal pain/cramping, and flatulence) after 4 weeks of treatment. Importantly, the majority of the participants in this trial reported some improvement in symptoms after only 1–2 weeks of treatment, with progressive improvement to the end of the study after 4 weeks (see Figure 5). This quick response is consistent with the proposed mechanism of action of silicolgel, which acts by adsorbing irritant molecules, preventing them from interacting in the gut, and would be expected to commence as soon as therapy begins.

### 4.3. Quality of Life

Bowel frequency is a key determinant of QoL in many people with IBS [14]; thus, therapies that can relieve diarrhoeal symptoms and abdominal discomfort have the potential to provide significant clinical benefits to individuals with IBS-D and IBS-M. People with these subtypes experience a greater impact on QoL than those with other IBS subtypes [14], as symptoms of diarrhoea can be debilitating and limit an individual's ability to socialise and go to work due to fear of bowel urgency and incontinence [16]. In this silicolgel study, the maximum frequency of bowel motions per day was significantly reduced, and the minimum frequency of bowel motions was reduced to a mean of 1.1 per day which many would consider “normal” after 4 weeks of treatment (see Table 3). The simplified QoL questionnaire was used before, during, and after treatment, and improvements in QoL were noted after 1 to 2 weeks of treatment, which is important not only to encourage continued treatment for the full duration but also to help boost confidence in control of bowel function.

### 4.4. Safety and Tolerability

Silicolgel was found to have a favourable safety profile, and all adverse events considered by investigators to be product-related were mild and were also consistent with symptoms of irritable bowel syndrome. This good tolerability profile is consistent with the literature on enteroadsorbents [23, 26] and the excellent safety-in-use data for silicolgel, which has been marketed as a medical device in the UK since 2014 and for longer in some European countries.

### 4.5. Comparison with Other Enteroadsorbent IBS Studies

The Howell et al. study in 2020 [23] is one of the few contemporary silica-based enteroadsorbent studies. It is difficult to directly compare data as the Howell study was a placebo-controlled trial with an 8-week double-blind phase, followed by an 8-week open-label phase. Also, the Howell trial focused on diarrhoea and abdominal pain as their primary response rates in IBS-D sufferers. However, the Howell study did report some IBS SSS data and overall patient “adequate relief of IBS” which can be compared to response data from this silicolgel study.

During the double-blind phase of their study, the Howell trial reported mean IBS SSS across weeks 5 to 8 of enteroadsorbent treatment which reduced from 334.4 to 184.5 (change: -149.9). Over the same time period, mean IBS SSS decreased from 352.3 to 240.8 (change: -111.5) for their placebo [23]. The mean IBS SSS for silicolgel after 4 weeks of treatment reduced from 323.4 to 160.3 (change: -163.1) which is similar to the IBS SSS reduction observed by Howell et al. for their enteroadsorbent during the 5–8-week period.

Furthermore, in the open-label phase of the Howell study, 75.9% of participants reported “adequate relief of IBS” after treatment with their enteroadsorbent [23], which is similar to the 83.6% response rate (IBS SSS reduction > 50) observed in this silicolgel study.

### 4.6. Comparisons with Other Current IBS Therapies

If standard dietary advice does not provide relief of IBS symptoms, then restriction diets including diets low in highly fermentable oligo-, di-, and monosaccharides (FODMAP) may be utilised. A meta-analysis of seven RCTs demonstrated that low FODMAP diets can reduce global symptoms in IBS compared to control, although the effect size was reduced when only RCTs with rigorous control diets were included [42]. Current evidence for the efficacy of low FODMAP diets is considered to be of “very low” quality according to GRADE criteria [42], and the generalisability of results from strictly controlled clinical trial environments with specialist dietary input to real-world practice may be limited.

First-line pharmacotherapy for IBS-D and IBS-M is frequently the over-the-counter (OTC) opioid agonist loperamide. Limited data from two trials with 42 patients indicates that loperamide therapy improves stool frequency and consistency but has no overall effect on global symptoms [43]. Furthermore, the use of loperamide in clinical practice can be limited by common adverse effects such as abdominal pain, bloating, and constipation [1]. Antispasmodic drugs such as mebeverine and hyoscine are widely available OTC and can reduce abdominal pain and global symptoms. However, there is considerable heterogeneity between trial results, and adverse effects such as dry mouth, visual disturbance, and dizziness can limit tolerability [43]. Peppermint oil is another OTC remedy which has demonstrated improvements in global IBS symptoms and abdominal pain compared to placebo, although evidence is considered to be of low quality and differences between formulations used in trials limit the generalisability of results [44].

Second-line pharmacotherapy may include gut neuromodulators such as tricyclic antidepressants and selective serotonin reuptake inhibitors which improve IBS symptoms and pain through their ability to modulate the gut-brain axis and alter transit time [43, 45]. Limitations of these medications include the need for dose titration, bothersome adverse effects such as drowsiness and dry mouth, and limited evidence of an effect on stool pattern [1, 43]. Antagonists of the 5-HT_3_ receptor, such as alosetron and ondansetron, are licenced for use in IBS-D and may slow gastrointestinal transit time and reduce visceral hypersensitivity [46]. Efficacy against placebo has been demonstrated for global symptoms, abdominal pain, and stool consistency [47], although treatment is generally restricted to secondary care and the availability in the community for this indication is limited. The nonabsorbable antibiotic rifaximin has also demonstrated efficacy against placebo in IBS-D in RCTs [47]. Issues with the cost and availability of this medication limit its utility in primary care settings.

Although multiple therapies with efficacy exist for IBS, evidence from meta-analyses is frequently assessed as low quality [1, 43]. Pharmacotherapy options are somewhat limited by adverse effects, unproven efficacy on certain disease parameters, and lack of availability in primary care settings where the majority of the burden of disease exists. Early evidence [22, 23] including the results of this study indicates that silica-based enteroadsorbents can demonstrate clinically meaningful improvements in abdominal pain, stool frequency, and global IBS symptoms with a favourable safety and tolerability profile. Given the current availability of enteroadsorbents in community settings in many countries, they represent a promising therapeutic option in this area of unmet need.

### 4.7. Study Limitations

The lack of a blinded placebo control arm is a limitation of this post marketing surveillance study. The large effect size seen accounts for both product efficacy and the placebo effect which can be substantial in IBS trials [22, 23, 48]. The development of adequately blinded placebos for gel-based products in gastrointestinal trials is challenging as it requires the use of thickeners, and these may have activity in the gut. Additionally, the trial was of short duration at four weeks to meet the regulatory recommended product treatment period. Therefore, the longer-term assessment of silicolgel was not possible, and the placebo effect might be expected to be greater in the early stages of treatment and wane over time. Another limitation is the use of a nonvalidated quality of life assessment; for practical purposes, a simplified assessment was used in place of the standard 34-question IBS QoL developed by Andrae et al. [41]. Finally, our trial is limited in size with 67 participants at a single site, and larger trials in a broader population are required to confirm the efficacy profile of silicolgel.

### 4.8. Summary

In this open-label study on silicolgel, a validated symptom assessment measure was used for the primary outcome, with results demonstrating a clinically meaningful impact (overall reduction in IBS SSS > 50) over the 4-week study period and with significant reductions in stool frequency. Secondary endpoints of reduction in self-assessed IBS symptoms and improvement in QoL support the primary outcome. Furthermore, the positive clinical benefits observed with silicolgel are consistent with those recently reported for another silica-based enteroadsorbent [23].

Further studies on silicolgel are planned, including placebo-controlled efficacy studies and in-vitro analyses to investigate the mode(s) of action of silicolgel.

## 5. Conclusions

Although IBS represents the most common gastrointestinal disorder seen by clinicians in both primary and secondary care [1], there is an unmet need for safe and effective therapies [19]. Enteroadsorbents have been used for many years for general gastrointestinal disorders, but clinical evidence for their efficacy in IBS and their use by physicians as part of IBS treatment has been limited.

The results from this open-label, observational study provide evidence that silicolgel is an effective and well-tolerated option for IBS sufferers with IBS-D and IBS-M when used in “real-life practice.” Clinically significant improvement in IBS SSS was demonstrated, with progressive improvements in IBS symptoms, QoL, and other outcomes.

This study adds to the body of evidence that enteroadsorbent therapy for IBS is effective and may provide a clinically beneficial treatment option for people with IBS-D and IBS-M. More studies to explore these promising results are warranted, including a randomised, placebo-controlled, double-blind trial, with longer-term follow-up, to further examine the efficacy of silicolgel in IBS.

## Figures and Tables

**Figure 2:**
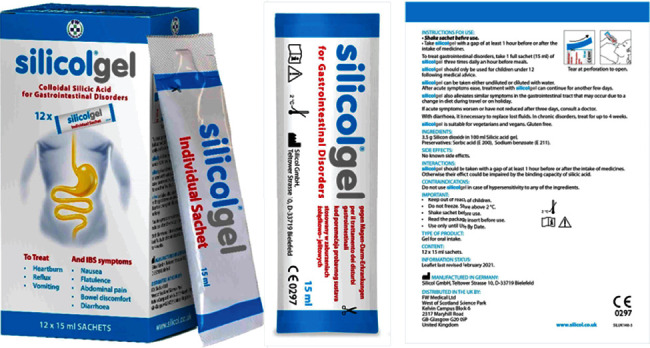
Silicolgel.

**Table 1:** Full exclusion criteria.

	Any of the following criteria excluded subjects from study participation:
1	Having a significant acute or chronic coexisting illness (cardiovascular, endocrine, immunological, metabolic, or any condition which contraindicates, in the investigator's judgment, entry into the study).
2	Having a diagnosis of inflammatory bowel disease, bowel cancer, coeliac disease, or any other bowel disease, which, in the opinion of the investigator, makes them unsuitable for entry into the study.
3	Any GI-related abdominal surgery other than hernia repair or appendectomy. Cholecystectomy more than 6 months previously is not an exclusion.
4	Individuals who have taken any antibiotics or oral steroids in the last 3 weeks.
5	Individuals planning to start any new medications during the course of the study
6	Known HIV infection, or hepatitis A, B, or C active infection.
7	Change in dose or introduction of an antipsychotic within the last month.
8	Have suffered from an uncontrolled or current major psychiatric disorder.
9	Individuals who, in the opinion of the investigator, are poor attendees or unlikely for any reason to be able to comply with the study requirements.
10	Subject is currently enrolled in or has not yet completed at least 30 days since ending another investigational device or drug study(s), or the patient is receiving another investigational agent(s).
11	Females who are pregnant or breastfeeding.
12	Unwilling or unable to comply with the study procedures. This includes leaving at least one hour between the ingestion of silicolgel and any other medication.
13	Taking a probiotic supplement and being unwilling to leave at least 1 hour between ingestion of IP and probiotic.
14	History of regular use of silicolgel or enterosgel for IBS.

**Table 2:** List of concomitant medications with efficacy in IBS.

		Pretreatment (visit 1) *N*=	After treatment (visit 4) *N*=
Use for IBS	Class		
Hyoscine butylbromide	Antispasmodic	10	2
Mebeverine	Antispasmodic	11	4
Alverine	Antispasmodic	1	0
Loperamide	Antidiarrhoeal	7	0
Ispaghula husk	Laxative	2	2
Docusate sodium	Laxative	2	1
Bisacodyl	Laxative	2	2
Probiotic	Probiotics	1	1
Colpermin	Peppermint oil	2	1

Use for non-IBS indications	Indication(s)		
SSRI	Depression/anxiety	16	16
Amitriptylline	Pain/migraine	2	2
Codeine-paracetamol	Pain	2	2
Gabapentin	Pain	2	2
Morphine	Pain	1	1

*N* = number of participants.

**Table 3:** Frequency of bowel motions, ITT, and by IBS type.

Study population	Mean daily bowel movements before treatment (visit 1)		Mean daily bowel movements after treatment (visit 4)	
	Maximum	Minimum	Maximum	Minimum
Overall (*n* = 67) *p* value for change	4.17 ± 2.19	1.56 ± 1.29	2.74 ± 1.65	1.12 ± 0.91
			*p* < 0.001^∗^	*p* < 0.03^∗^

IBS-D (*n* = 37) *p* value for change	4.69 ± 2.24	1.82 ± 1.29	3.08 ± 1.58	1.35 ± 0.87
			*p* < 0.001^∗^	*p* < 0.08

IBS-M (*n* = 30) *p* value for change	3.52 ± 1.98	1.24 ± 1.28	2.33 ± 1.66	0.83 ± 0.90
			*p* < 0.02^∗^	*p* < 0.20

Values are given as mean ± standard deviation. IBS-D = IBS with predominant diarrhoea; IBS-M = IBS with mixed bowel habits. ^∗^*p* values < 0.05 are considered statistically significant.

**Table 4:** Baseline characteristics of study population.

Characteristic	Value
Overall cohort	67
IBS-D subtype	37 (55%)
IBS-M subtype	30 (45%)
Age	40.9 (20-63)
Female (%)	59 (88%)
Body mass index	27.7 (18.6-39.0)
IBS severity (by IBS SSS score)	
Severe (IBS SSS > 300)	42 (63%)
Moderate (IBS SSS 175-299)	25 (37%)
Mild (IBS SSS 75-174)	0 (0%)
Baseline use of concomitant IBS medications	
Antispasmodic	21 (33%)
Antidiarrhoeal	7 (10%)
Laxative	6 (9%)
Other	3 (4%)

Values are given as the mean (range) or the number (percentage of the cohort). IBS = irritable bowel syndrome; IBS-D = IBS with predominant diarrhoea; IBS-M = IBS with mixed bowel habits; IBS SSS = irritable bowel syndrome severity scoring system.

**Table 5:** IBS SSS before and after treatment, ITT, and by IBS type.

Study population	IBS severity scoring system (IBS SSS)				Responders (%)
	IBS SSS visit 1 before treatment (baseline)	IBS SSS visit 4 after treatment (4 weeks)	Mean change in IBS SSS score	*p* value	
Overall (*n* = 67)	323.4 ± 55.7	160.3 ± 90.3	**-163.1**	*p* < 0.001	**56** (83.6%)
IBS-D (*n* = 37)	319.3 ± 53.7	155.8 ± 98.4	**-163.5**	*p* < 0.001	**30** (81.1%)
IBS-M (*n* = 30)	328.6 ± 58.6	166.0 ± 80.5	**-162.6**	*p* < 0.001	**27** (90.0%)

IBS = irritable bowel syndrome; IBS-D = with predominant diarrhoea; IBS-M = with mixed bowel habits. Values are supplied as mean ± standard deviation. The mean reduction in IBS SSS after 4 weeks of treatment is significant (*p* < 0.001). Subjects with a reduction in IBS SSS of >50 from baseline at visit 4 were considered responders. Mean reduction in IBS SSS ≡ change from severe IBS to mild IBS on Francis et al. scale [35].

**Table 6:** Mean IBS symptoms severity scores: before and after 4 weeks of treatment, total ITT, and by IBS type.

Study population	IBS symptom scores: 0 = not bother me at all, 10 = as bad as I can imagine								
		Diarrhoea		Abdominal pain/cramping		Bloating		Flatulence	
		Visit 1	Visit 4	Visit 1	Visit 4	Visit 1	Visit 4	Visit 1	Visit 4
Overall (*n* = 67)	Mean ± SD	7.24 ± 1.79	3.31 ± 2.43	7.28 ± 1.50	3.21 ± 2.54	6.72 ± 2.61	3.37 ± 2.53	6.82 ± 2.32	3.37 ± 2.55
	Change		*p* < 0.001		*p* < 0.001		*p* < 0.001		*p* < 0.001

IBS-D (*n* = 37)	Mean ± SD	7.49 ± 1.50	3.75 ± 2.81	7.03 ± 1.74	3.11 ± 2.67	6.77 ± 2.77	3.25 ± 2.69	6.89 ± 2.41	3.42 ± 2.61
	Change		*p* < 0.001		*p* < 0.001		*p* < 0.001		*p* < 0.001

IBS-M (*n* = 30)	Mean ± SD	6.93 ± 2.07	2.86 ± 1.79	7.60 ± 1.07	3.41 ± 2.47	7.27 ± 2.33	3.62 ± 2.41	6.73 ± 2.24	3.38 ± 2.56
	Change		*p* < 0.001		*p* < 0.001		*p* < 0.001		*p* < 0.001

Values are given as mean ± standard deviation. IBS-D = IBS with predominant diarrhoea; IBS-M = IBS with mixed bowel habit. Change = *p* value for difference between visit 1 and visit 4. For all 4 key IBS symptoms, the mean severity scores showed a significant reduction after 4 weeks of treatment. Note. Data is available for telephone visits 2 and 3 (one week and two weeks of treatment, respectively) which shows fast and progressive improvement in all 4 symptoms across the duration of the study.

**Table 7:** Adverse events listing.

AEs by system organ class	*N*=	Causality assessment	
		Not related to treatment (*N*=)	Related to treatment (*N*=)
Gastrointestinal disorders			
Diarrhoea	4	2	2
Dyspepsia	5	3	2
Gastroenteritis	1	1	
Constipation	3		3
Abdominal pain	2	1	1
Anorexia	1		1
Stool urgency	2		2
Vomiting	1	1	
Infections and infestations			
Upper respiratory tract infection	7	7	
Flu-like illness	1	1	
COVID-19	2	2	
Lower respiratory tract infection	2	2	
Urinary tract infection	2	2	
Psychiatric disorders			
Emotional stress	1	1	
Reproductive system and breast disorders			
Menorrhagia	1	1	
Dysmenorrhoea	1	1	
Nervous system disorders			
Headache	4	4	
Blood and lymphatic system disorders			
Anaemia	1	1	
General disorders and administration site conditions			
Malaise	2	2	
Musculoskeletal and connective tissue disorders			
Shoulder tendonitis	1	1	
Ear and labyrinth disorders			
Bilateral ear infection	1	1	
Serious AEs			
Optic neuritis	1	1	
Vomiting	1	1	

AE = adverse event; *N* = number of participants.

## Data Availability

The data that support the findings of this study are available from the corresponding author upon reasonable request.
