# Correction to “Efficacy of the Enteroadsorbent Silicol®gel in Adults with Irritable Bowel Syndrome Subtypes IBS-D or Mixed: Observational Open-Label Study”

**DOI:** 10.1155/grp/9856930

**Published:** 2025-10-03

G. Crawford, R. Taylor, D. Young, and C. G. Hatton, “Efficacy of the Enteroadsorbent Silicol®gel in Adults with Irritable Bowel Syndrome Subtypes IBS-D or Mixed: Observational Open-Label Study,” *Gastroenterology Research and Practice* 2023 (2023): 3432763, https://doi.org/10.1155/2023/3432763.

In the article titled “Efficacy of the Enteroadsorbent Silicol®gel in Adults with Irritable Bowel Syndrome Subtypes IBS-D or Mixed: Observational Open-Label Study” there was an error in Table 5. The value in the bottom right column of the table read: 27 (90.0%).

The correct value is 26 (86.7%). The corrected table is shown as follows and is listed as Table 5.

The corrected section appears as follows:

We apologize for this error.

## Figures and Tables

**Table 1:** IBS SSS before and after treatment, ITT, and by IBS type.

**Study population**	**IBS severity scoring system (IBS SSS)**				**Responders (%)**
	**IBS SSS Visit 1 before treatment (baseline)**	**IBS SSS Visit 4 after treatment (4 weeks)**	**Mean change in IBS SSS score**	**p** ** value**	
Overall (*n* = 67)	323.4 ± 55.7	160.3 ± 90.3	**−163.1**	*p* < 0.001	**56** (83.6%)
IBS-D (*n* = 37)	319.3 ± 53.7	155.8 ± 98.4	**−163.5**	*p* < 0.001	**30** (81.1%)
IBS-M (*n* = 30)	328.6 ± 58.6	166.0 ± 80.5	**−162.6**	*p* < 0.001	**26** (86.7%)

*Note:* Values are supplied as mean ± standard deviation. The mean reduction in IBS SSS after 4 weeks of treatment is significant (*p* < 0.001). Subjects with a reduction in IBS SSS of > 50 from baseline at Visit 4 were considered responders. Mean reduction in IBS SSS ≡ change from severe IBS to mild IBS on Francis et al.'s scale [35].

Abbreviations: IBS, irritable bowel syndrome; IBS-D, with predominant diarrhea; IBS-M, with mixed bowel habits.

